# Operations for the Cure of Stammering

**Published:** 1841-08

**Authors:** 


*Operations for the Cure of Stammering.*—Our last files of foreign journals are filled with animated discussions, and reports of the transactions of medical societies, in relation to the several new operations proposed for the cure of stammering. The operations are those of Yearsley, Lucas, and Dieffenbach: each founded upon a different principle; and yet each professing to accomplish a cure in the majority of cases. Mr. Yearsley gives the following account of the manner in which he was led to the discovery of the operation which he recommends:

"In the practice of my department of the profession, it has been usual with me to explore the condition of the mouth and pharynx in every case of deafness committed to my care. I have thus discovered that a large number of patients suffering from deafness, are affected with enlargement of the tonsils and uvula, and an irritable condition of their investing membrane and the pharynx generally. It has been my constant practice, when I have considered these states at all contributing to the imperfection in hearing, to remove either the tonsils or the uvula, or portions of both, according to the nature of the case, with the most marked and immediate benefit, as far as the hearing may have been concerned. In December last it occurred to me to operate in this manner on two patients. They were, at the time of treatment, so deaf, that I did not then address my questions particularly to them, but to their parents, so that I was unaware of any impediment to speech in these instances. Some time after, as the cure of deafness advanced, I learned from the parents that both children had been stammerers from infancy, and, as much to my surprise as gratification, that the cure of stammering had ensued immediately on the excision of the tonsils. At the time at which I write, the subjects of both these cases remain free from any impediment, though their stammering previously to the operation is represented to me as having been very decided. I had before this remarked that persons with enlarged tonsils were affected with thick and imperfect speech, for which I had often, during the last year, practised excision with the happiest effect, in restoring the voice to its original clearness. Since the cases above mentioned, I have operated on upwards of forty persons, all of whom have immediately felt themselves relieved of their impediment. Many have seemed wild with joy, or have shed tears of pleasure, at the instantaneous restoration they have enjoyed. After the operation, the difficulty of speech which remains is referred by the patient to the lips; they express themselves entirely free from the original difficulty. Something must be allowed for the long misuse of the organ of voice, and the existence of habit, in rendering the voice less perfect than in a natural state. In fact, after their relief, patients have yet to learn the proper use of the vocal apparatus.

"I have performed the operation by means of a scalpel, tenaculum, and scissors, without any serious hæmorrhage, and with a small amount of pain, which has appeared somewhat greater in the case of the uvula than the tonsils.

"In reflecting upon the subject, the explanation I have at present to offer is, that to produce stammering, the dorsum linguæ, the palatine arches, velum palati, and uvula, approximate together so completely, and perhaps irregularly, as to leave no room for the expulsion of air from the larynx. In a person who stammers, no air issues from the mouth during the abortive effort to speak ; but it does so as soon as the patient is relieved from this state, so as to produce sound. The most powerful contraction of the abdominal muscles can be seen attempting to force up the diaphragm and expel the air; sometimes all the respiratory muscles, and even those of the body generally, are thrown into violent spasmodic action, as the individual grasps some near object to assist the expulsive effort. In some cases, when there is nothing abnormal about the tonsils or uvula, I find a great congenital narrowing of the entrance from the mouth to the pharynx."

Mr. Yearsly, it appears, has operated upon one hundred and twenty patients, of whom only three were women ! the great majority of these were cured, all were more or less relieved—so say Mr. Yearsley's partisans. The London Medical Society, however, openly condems every operation, because "stammering is essentially functional," and the operations in question "are not based on any scientific basis." While the Westminster Medical Society admitting, what certainly cannot be disputed, that real cures have *occasionally* followed these attempts—particularly those of Mr. Yearsley, which are the most numerous,—starts from this point as a fact, and occupies itself more usefully in endeavoring to trace out the relation between cause and effect. The following is a brief summary of the opinions expressed by some of the distinguished members in reference to the mode of action of the operation, condensed from the Lancet.

"With regard to the extent of the operation, Mr. Yearsley, in most cases, removed the uvula entirely, chiefly with the view of throwing both arches of the palate into one. When the tonsils were so much enlarged as to project beyond the columns of the fauces, the uvula was then partially removed, and also so much of the tonsils as projected; in only one case had any thing like severe hæmorrhage took place, and in this the bleeding continued for four hours. The operation, however, might not be unattended by danger, when there was a diathesis to hæmorrhage present in the system."

Mr. Dowing, who had witnessed several of the operations, and was struck with the fact that three methods so entirely different in their character were all followed in the majority of cases by success, was disposed to consider the effect dependent on the shock to the nervous system, and the loss of blood.

Mr. Alcock also had seen many operations performed by Mr. Yearsley. Some of these were attended with *marvellous improvement*, others with great relief, while others were productive of no benefit—believing that stammering depended on a variety of causes, he could not conceive that Mr. Yearsley's method would be applicable to all cases, but had no doubt that in many it would be followed by a permanent cure.

Mr. Malyn had been present at three operations, all of which were successful, although, only in one instance, did the uvula upon inspection appear unnaturally elongated. Mr. Malyn believes that stammerers experience the greatest difficulty in uttering the labial or dental sounds, because in the effort the velum descends and hangs over the windpipe, thus preventing a sufficient supply of "material for the manufacture of words;" by the operation of Mr. Yearsley this impediment is removed, the supply of material is rendered sufficient and the stammer ceases.

The opinion of Dr. Marshall Hall is more at length and more scientific; we quote it in his own words:

"He observed, that the previous speakers had not, he thought, entered into the true line of argument. Too much had been said about the tumidity of the tonsils, and of the tongue; too little about the functional properties of the organs of articulation. Now, tumidity of the tonsils induced a certain well-known and equally recognisable thickness of the voice; tumidity of the tongue induced a special defect of articulation, but neither of these induced stammering. On the other hand, stammering was excited in a little patient of his whenever the general health was deranged. Dr. Bostock has detailed, in the "Medico-Chirurgical Transactions," a case of stammering cured by the administration of purgative medicines; and, lastly, stammering was excited as a part of chorea. These, and other facts, proved that stammering was not so much an organic as a functional defect; and the question to be agitated that evening was, what relation the excision of the uvula could have with the cure of such a malady. Dr. Hall had been witness to two cases: the first was that of Philip Wyatt; before the operation, which consisted in the removal of the uvula, the patient was asked his name; in vain he attempted to enunciate the Ph; the effort seemed to threaten convulsions; the operation was performed; the same question was put, and the ready reply was, Philip Wyatt! In the second case the good effect, though less complete, was not less obvious. Now, what could be the rationale of this phenomenon? That it could be at all connected with the more or less open state of the air-passages, Dr. Hall regards as most improbable. In many cases there was no enlargement of the tonsils or tongue, no elongation of the uvula, neither was there any want of volume or force of the expired air, when the word was well pronounced, or in the pronunciation of such letters as did not absolutely interrupt the flow of the expired air, as v., s., &c.; nor was there, as Dr. Arnott thought, any obstruction to the flow of air in the larynx itself, as he (Dr. Hall) had shown in a paper published in the "Journal of the Royal Institution," in 1831. The obstruction was offered by the organs, not of the voice, but of the articulation; not of the physical condition of the parts, but of their undue action. In pronouncing the letter b. the mouth was closed by the force on adduction of the lips, the posterior nares being closed by the veil of the palate. Long ago Dr. Hall had described stammering as an undue spinal action; the stammering of chorea proved this. He would now venture to ask, might the situation of the uvula and its peculiar contact with the parts of the posterior nares lead to a reflex spinal action? The act of vomiting on tickling the fauces was not less marvellous or inscrutable ; the uvula in this manner might be the excitor and regulator of speech. Stammering might be induced in cases in which its posterior surface was unduly excitable. In this manner we might explain the effects of Mr. Yearsley's operation ; but Dr. Hall begged the society to view his last observation as a conjecture. Elongation of the uvula could have no effect in inducing stammering as supposed, by falling on the tongue ; for in the enunciation of many letters, as b, t, v, s, &c., it was raised, with the velum, high up, so as to assist in closing the posterior nares. Time and further investigation were, as Mr. Yearsley was aware, required to mature the investigation, to determine the special cases of stammering to which the operation was adapted. But enough had been done to excite the deepest interest in every liberal mind."

Mr. Robins had witnessed several operations by Mr. Y., attended by such success as would induce him, although he believed stammering to depend on a variety of causes, to recommend to every one afflicted with this defect to submit to the removal of his uvula.

In a word, the conclusions to be drawn from the discussion before the Westminster Medical Society are as before stated. That the operations are occasionally followed by an entire and permanent cure, more frequently are attended by relief—the relief consisting in the removal of the spasm by which the stammer is accompanied, rather than in any benefit to the stammer—while in other cases the operation effects no change.

A question of preference however exists between the operation which we have just noticed and that recommended by Messrs. Lucas and Amusat. This latter consists in a division of the frœnum, and a removal of a portion of the anterior fibres of the genio-hioglossi muscles ; the relative advantages of these two methods, and the particular cases to which each is applicale, remain unsettled.

In order to complete the history of this subject as at present before the profession, we have only to introduce the more dangerous and difficult operations devised by Dieffenbach, which we extract in the words of his translator, Mr. Travers.

"The idea lately suggested itself to me," say Dieffenbach, " that an incision carried completely through the root of the tongue might possibly be useful" in stuttering, which had resisted other means of cure, "by producing an alteration in the condition of its nervous influence, allaying the spasm of the chordæ vocales, &c." A most unphilosophical beginning, truly, and one which speaks strongly of the discipline of the German hospitals, and possibly of the little danger of an inquest on unsuccessful practice. One thing is certain, that an idea so conceived would be regarded as most unwarrantable in its application in the British hospitals. Nor is the case improved, when he explains that the first thought of the operation originated in a patint with strabismus one day requesting to be operated on for that deformity, with a "well-marked stutter." But he continues—"the brilliant success of this new oparation more than realized my most sanguine expectations."

The principle which the professor has in view, is the division of the nervous filaments distributed to the muscular substance of the tongue, under the impression that some undue excitation in these nerves induces the spasmodic movement of the muscles. That this effect may result from the operation is more than probable; but it appears to us that the method of arriving at the end in view is exceedingly clumsy. If mere alteration of the innervation of the muscles of the organ be the object, why not, by a much more simple, incomparably less severe, and less dangerous operation, at once divide the hypoglossal nerve or nerves as they rest on the hyoglossus muscles; an operation requiring a superficial incision of scarcely an inch in length.

The author prescribes three methods of operating. "1. The transverse horizontal division of the root of the tongue. 2. The subcutaneous transverse division, in which the mucous covering of the tongue is left inviolate. 3. The horizontal division with excision of a wedge-shaped portion."

"The first operation I performed on the 7th of January, 1841. I chose for this case the method by which a wedge-shaped portion is removed from the posterior part of the tongue; for, as I have remarked, I felt more confidence in this than in the other methods.

"Frederick Doenau, a highly intelligent and talented boy of thirteen years of age, had stuttered from his earliest childhood, and to so painful an extent, that the defect was thought to be quite incurable. It varied, however, much in degree: when at the worst, he was unable even to produce a sound. He stuttered in Latin and French, as well as in his own language—sometimes one set of words, and sometimes on others. The pronunciation of the sibilant letters (s, z, ss,) and of the palatals hard (g, k, ch, and x,) was attended with particular difficulty; and he made no distinction between the hard sounds, p, t, k, and the soft ones, b, d, g, (German.) He repeated the same letter often four times running; and when he whispered, he stuttered as much as when he spoke loud or shouted; often he could either not speak at all, or produced only half articulate sounds. The presence of a stranger invariably affected him in a manner most painful to behold. His face became distorted; the alæ of the nose worked convulsively; his lips moved quiveringly up and down; his eyelids were expanded into a wild and eager stare; the tongue was now stiff, now played convulsively within the mouth; and the muscles of the throat, larynx, and trachea were sympathetically affected. Thus, after terrible efforts, the boy gave utterance to a mangled and imperfect word; now for a time was his speech free, and words chased one another with incredible velocity, till confusion ensued amidst the thronging sounds; and the same painful scene was thus again and again renewed. The peculiar physical horror which constitutes a stutterer, and which is excited by the effort to speak, is very similar to that which gives rise to the excitement and spasm of the hydrophobic patient at the sight of water. This internal movement might, on that account, be called phonophobia.

"The boy's mother caught eagerly at my offer to make an effort to cure him; accordingly, with the assistance of Drs. Holthoff and Hildebrant, the operation was performed as follows: The boy sat with his head leaned against the breast of an assistant; the tongue being protruded as far as possible, was grasped on its anterior half with the forceps of Muzeux, being thus compressed latterly, and drawn forwards by one assistant. The gentleman against whose breast the boy's head rested, retracted the angles of the mouth with a pair of blunt hooks. Grasping now the tongue as near to its root as possible, between the thumb and forefinger of the left hand, I passed the bistoury through it, and divided it completely from below upwards; a strong ligature passed through the posterior edge of the wound, served to fix it temporarily, and prevent too great a strain upon the slender band which alone connected the mass of the tongue to it; the anterior lip of the incision was now grasped, and laterally compressed between the modified hare-lip forceps, and a wedge-shaped slice excised out of the whole thickness of the tongue. It will be found more convenient to make this second incision from above downward, and with a small straight knife. The posterior edge of the wound was now, by means of the beforementioned ligature, and a sharp double hook, drawn so far forwards that the needles with the ligatures could be conveniently passed through it; six strong sutures served to bring the edges of the wound together, and to restrain the hemorrhage. To effect the latter object, they must include the whole depth of the wound within their loop. That the hæmorrhage was considerable, may be imagined from the nature of the operation, which should not be attempted by all persons indiscriminately. As soon as the boy's mouth was washed out, I desired him to pronounce some of those words which he had before found especially difficult; he did so without stuttering or hesitation. This distortion of the face, however, continued; the patient was put to bed, and a cooling plan of treatment ordered. With the exception of a slight sympathetic febrile disturbance, the swelling of the tongue, that one might anticipate, and the consequent impeded deglutition, nothing remains to be noticed, so far as regards his recovery from the operation itself. His features, and his mouth especially, were still much distorted when he spoke, but the stutter had entirely ceased. On the fifth day I removed three of the sutures; during the next twenty-four hours the swelling of the tongue had visibly decreased, and I then removed the three remaining sutures. On the seventh day the wound was completely healed, the back part of the tongue alone was very inconsiderably swelled, and the boy quite re-established. At this present time, not the slightest trace of stuttering remains, not the slightest vibration of the muscles of the face, not the most inconsiderable play of the lips. His speech is throughout clear, well-toned, even, and flowing. Neither inward emotions nor unexpected external impressions, produce the slightest hesitation; he can speak, read, entertain himself indifferently with friends and strangers."

"The total number of stutterers that I have relieved up to this time, is sixteen, and those who are as yet under treatment appear to promise equally favourable results."

The following paragraph is deserving of serious consideration, not less on the part of those afflicted with the serious inconvenience in question, but also by the operator. We are informed that in an operation for stuttering, performed a short time since in London, the hæmorrhoge was fearful.

"In this operation it is more difficult to prescribe for the individual modifications of each particular case, than in the operation for strabismus, and it can never be performed by one who has not the temperament of an operator: the hæmorrhage must hold all others at a respectful distance. The extent and importance of the operation, the possible danger to life, or loss of the tongue, either through the want of skill in the assistants, who may tear it off when so nearly separated, or through mortification or ulceration of its connecting isthmus. These are contingencies rationally to be feared, and which must be carefully weighed beforehand."

Professor Dieffenbach concludes with a few words of criticism upon the herd of imitators who follow in the wake of talent, which are worth perusing, especially as they are evidently intended as a satire upon the instrument-discoverers, and new-method incubators of squint-cutting. Thus he observes,

"Amidst the prevailing rage for modifying operations, I foresee that my having described the three principal available methods, cannot fail to open to surgeons a vast field for the discovery of modifications, and the creation of instruments. We shall have conical and oblique incisions, from the surface and under the skin! Actual and potential cautery! We shall have knives and scissors with improved curves, and a thousand variously-fashioned forceps and hooks. They will set the blades at angles with the handles to allow a better light falling into the mouth. Opportunity is likewise afforded to professional antiquaries to hunt after a name for this operation. To them I freely make over the right of baptism."

To this we may add a case reported by M. Franz, of London, who performed Dieffenbach's third operation, viz., the excision of a wedge from the root of the tongue on the first of March.

The patient (who was a young man aged 17) bore this severe operation well, but became rather faint towards the termination, and afterwards vomited large quantities of blood which he had swallowed. As soon as he had washed his mouth with a little water, I was exceedingly pleased to hear him pronounce words which, previouly to the operation, he was utterly unable to articulate; such as time, powder, &c.; without the slightest hesitation or stammering, and without any twitchings of the lips, or even convulsive movements of the muscles of the face or neck, and immediately afterwards I was surprised by his saying with facility and distinctness, "there is some blood running down my shirt." He was now put to bed, and desired to keep quiet, and directed not to be allowed to speak, and to have his mouth kept cool by means of cold water. On my calling, in two or three hours time, I found the case proceeding favourably; no reaction had as yet taken place.

"March 9th.—Has for the first time taken a walk in the open air. The movements of the tongue less painful. The mother gives a favourable account of the progress of his speech.

"Perhaps it may be as well to state that the muscles divided in this operation, are the lingualis, the genio-hyoglossi, the hyo-glossi, and the stylo-glossi."

We here leave the subject without comment; experience alone can decide upon the merits, both positive and relative, of these different operations.—*Med. Examiner.*

